# Supplementary material for: Barriers and facilitators to HIV and viral hepatitis testing in primary healthcare settings in the Kyrgyz Republic: A mixed-methods study using the COM-B Framework
Source: PLoS One. 2025 Nov 17;20(11):e0336257. doi: 10.1371/journal.pone.0336257 (PMC12622783; doi:10.1371/journal.pone.0336257)
Supplement: S1 Appendix — (DOCX) [file pone.0336257.s001.docx]

Welcome and thank you for participating in this interview!

Your insights and experiences are incredibly valuable to our research. This interview is part of a study being conducted by the National Public Health Institute (Kyrgyzstan) and Robert Koch Institute (the National Public Health Institute in Germany) to identify and describe the most important barriers and facilitators to HIV and viral hepatitis B, C and D testing in primary health care settings in Kyrgyzstan.

The purpose of this interview is to collect information about your personal experiences, perspectives and thoughts regarding HIV and viral hepatitis B, C and D testing. There are no right or wrong answers, and your perspective will help us better understand the challenges and opportunities faced by healthcare professionals like you, and will contribute to the development of more effective and responsive public health interventions and policies.

My name is [your name] and I am going to conduct this interview with you today. Our interview will take approximately 1 hour. You may choose to skip any questions you are uncomfortable answering, and you may withdraw from the interview at any time without consequence. With your permission, we would like to record the interview to ensure that we capture your answers accurately. The recordings will be kept securely and used for research purposes only.

**Section 1: Opening questions**

Could you please describe your experience with providing healthcare services, specifically viral hepatitis testing?
- How would you describe you experience with HIV testing? *(depending on the answer to this the following question can be adapted)*

- How do you think viral hepatitis affect people's health in your country? What about HIV??

**Section 2: Testing Practices (by HCW in their setting)**

- Can you walk me through the steps for viral hepatitis testing in your facility? Is it the same for HIV? Are patients sometimes tested for both at the same time? (*integrated testing*)
- How is your experience with offering hepatitis tests test to patients? And what about HIV? *(depending on their answer to the first questions, consider adapting or even ask about integrated testing**)*
  - To which patients/in what situations would you offer a hepatitis test? What about HIV tests? *(**)*
  - Do you feel comfortable talking to patients about risk factors for infectious diseases such as HIV and viral hepatitis which warrants testing (reference to strategy)?
  - (*If not much experience/not offering tests*) What are the main reasons for not offering a test? *(consider asking only separately depending on answers above to HIV or VH or find out of more difficult with HIV compared to VH)*

*Probes*:

- - Only tests if patient asks to be tested
  - Lack of time?
  - Shortage of staff?
  - Lack of knowledge?
  - Does not want to deal with such patients?
  - Are there any other problems with how things are organized or set up? (for example, opening hours, lack of physical space (e.g. for pre-/test counselling), transport of samples etc.)?
  - Do not see it as their responsibility? (primary healthcare settings)
  - Other diseases “more” important?
- Are rapid tests available in your healthcare setting?
  - How would you describe the role of HIV/hepatitis (**) rapid tests in your setting?
- How is the follow-up with patients conducted post-testing, especially in cases of positive results (linkage to care/treatment pathway)?
- For those who are hepatitis B positive, are they offered hepatitis D testing? (**)
  - What is your personal experience with handling positive test results? *(See if they feel comfortable to talk about positive results with patients) (probe: ask something about if lack to access to treatment/not knowing what to do with a positive patient, does that sometimes prevent testing)*

**Section 3: Information on testing for the HCW**

- How did your (medical) studies prepare you for offering hepatitis testing? Is it the same for HIV?

*Probes*:

- - - - Need/role for additional training?
      - How often?
      - By whom?
- How do you keep yourself updated in terms of who to offer (HIV/hep) testing?

*Probes:*

- - Decrees, staff updates, written information from head of the clinic

**Section 4: Barriers to Testing**

- In your experience, what are the most important reasons why healthcare workers do not offer testing for hepatitis to patients?
- Do the same reasons apply for HIV? (**)
- Which factors influence attitudes towards testing among your colleagues?

*Probes:*

- - language
  - gender identity of doctors vs. Gender identity of patients
  - sexual identity of patients
  - lack of trust to doctors or low awareness
- Are there any key populations (*describe what we mean here by mentioning a few examples*) that have more difficulties accessing testing? Can you elaborate on why that might be the case? Are there any differences between HIV and viral hepatitis that you want to mention?

*Probes:*

- - People who use drugs?
  - Gay, bisexual and other MSM?
  - Trans* people?
  - Sex workers?
  - Working migrants or family members of working migrants
- In your opinion, how does the legal and policy environment towards key populations impact HIV and viral hepatitis testing?

*Probes:*

- - Free testing
  - LGBT propaganda law
  - Substance use is partially criminalized
- Do stigma and discrimination play a role in deterring individuals from getting tested? How is this addressed in your practice?

**Section 5: Facilitators for Testing**

- Have there been any initiatives, which improved testing rates for HIV and viral hepatitis (**) in your setting? Please elaborate.

*Probes:*

- - Free testing
  - Link from NGOs (training, information to patients from other organizations)
  - Support from government/international organizations
- What do you think could improve testing rates for HIV and viral hepatitis in your settings? *Probes:*
  - technological advancements?
  - Awareness raising among population? Among doctors?
  - Are there any differences for hepatitis and HIV that you want to mention?

**Section 6: Closing questions**

- Are there any aspects around HIV or hepatitis testing (**) that we did not cover that you would like to add?

**Thank you so much for your valuable time and for participating in this interview!**
