# Supplementary material for: Barriers and facilitators to HIV and viral hepatitis testing in primary healthcare settings in the Kyrgyz Republic: A mixed-methods study using the COM-B Framework
Source: PLoS One. 2025 Nov 17;20(11):e0336257. doi: 10.1371/journal.pone.0336257 (PMC12622783; doi:10.1371/journal.pone.0336257)
Supplement: S2 Appendix — (DOCX) [file pone.0336257.s002.docx]

Barriers and facilitators to HIV and viral hepatitis testing in primary healthcare settings in Kyrgyzstan

Welcome to the survey on barriers and facilitators to HIV and viral hepatitis testing in primary healthcare settings in Kyrgyzstan!

**KGZ:**To complete the questionnaire in Russian, please change language via the drop-down menu to the top right.

This study is carried out to obtain information on main barriers and facilitators for viral hepatitis and HIV testing as perceived by primary healthcare workers (HCW). In Kyrgyzstan, there is to date little information on main barriers and facilitators for testing among HCW, and this information will help the implementation of the new strategy on HIV and viral hepatitis elimination (2023-2027) in Kyrgyzstan with focus on increasing testing.

This study is being conducted by the National Public Health Institute and the Republican Center for Viral Hepatitis und HIV/AIDS Control at the Ministry of Health of the Kyrgyz Republic in collaboration with epidemiologists from the [Robert Koch Institute](https://www.rki.de/EN/Home/homepage_node.html) (RKI), the national public health institute in Germany.

**Participation in the survey is voluntary**. **All information that we collect for the study will be treated as strictly confidential**. The participation in the survey is **anonymous**, and with your participation you agree that your provided data in this survey may be used for research purposes only.

To help us to keep it anonymous, please make sure that any answers in free text fields do not contain any personal data about yourself or others (e.g. names, addresses or similar). Please answer all questions to the best of your ability, but if you do not wish to answer, you are free to skip a question. You can also end the survey at any time or withdraw your participation therein. There will be no negative consequences, if you decide to not take part in the survey. Each person should only complete the questionnaire once.

It will take about 15 minutes to complete the survey. If you have any questions or need for further information please contact: Dr. med. Zuridin Nurmatov (E-Mail: [zuridin@mail.ru](mailto:zuridin@mail.ru), Telephone: +996778100477) or Nikolay Lunchenkov (LunchenkovN@rki.de).

I hereby confirm that I am above 18 years of age and work as a primary healthcare provider in Kyrgyzstan. I have read and understood the conditions and I consent to participate in the survey:

Yes ( )

No ( )

Sociodemographic Information

*In the first part of the questionnaire, we would like to ask you a few questions about you and your work.*

- How old are you? (drop-down)
  - 30 years or younger
  - 31-40 years
  - 41-50 years
  - 51-60 years
  - 61+ years
  - Do not want to answer
- What is your gender? (drop-down)
  - Female
  - Male
  - Other
- In which region are you currently working as a primary healthcare practitioner? (drop-down)
  - Batken
  - Bishkek
  - Chui
  - Issyk-Kule
  - Jalal-Abad
  - Naryn
  - Osh City
  - Osh Region
  - Talas
- Is your place of work located in a city or a village? (drop-down)
  - City
  - Village
- What is your main healthcare specialty/background? *(possible to tick more than one option)*
  - Family medicine
  - Infectious Diseases
  - Dermatovenerology
  - Internal Medicine
  - [Obstetrics and Gynecology](https://www.aucmed.edu/about/blog/a-complete-list-of-medical-specialties-and-subspecialties#obgyn)
  - Other: Please specify: _______________

Specific questions regarding HBV, HCV and HIV testing

*In this second part of the questionnaire, we would like to ask you a few questions about HBV, HCV and HIV testing in your facility.*

- Which tests are available for free in your healthcare facility?

*(possible to tick more than one option)*

- - Hepatitis B tests
  - Hepatitis C tests
  - HIV tests
- Have you performed testing for hepatitis B, C or HIV during the last 12 months? *(possible to tick more than one option)*
- Yes, hepatitis B
- Yes, hepatitis C
- Yes, HIV
- No
- I don’t want to answer
- If yes, have you performed testing for hepatitis B, C and/or HIV at the same time for one patient (this is also known as integrated testing) during the last 12 months?
- Yes
- No
- If no, what were the main reasons:
  - I don’t know what integrated testing is
  - Patient presented with hepatitis symptoms, therefore I did not test for HIV
  - There were no tests available for integrated testing
  - I didn’t feel I had the knowledge to explain reasons for integrated testing to the patient
  - I don’t think integrated testing is a good approach for diagnosing more hepatitis B, C and HIV patients
  - Other: _________________
- Were there cases the last 12 months where you did not test a patient despite of clinical indication for testing* for either **HBV, HCV or HIV**?
  - Yes
  - No
  - I don’t remember
  - I prefer not to say

**should be tested according to the latest decree (number XX)*

Barriers and facilitators to testing

*Despite best intentions, there are many reasons for why testing might not always be offered to a patient. In the next section, please tick one box to each of the statements below to indicate which best describe barriers and facilitators for testing in your facility.*

- Lack of time makes it difficult to offer testing for HBV, HCV and/or HIV
  - Strongly disagree
  - Disagree
  - Neither agree nor disagree
  - Agree
  - Strongly agree
- We are too few doctors to be able to offer testing HBV, HCV and/or HIV
  - Strongly disagree
  - Disagree
  - Neither agree nor disagree
  - Agree
- We do not have enough physical space to offer confidential testing in our healthcare facility
  - Strongly disagree
  - Disagree
  - Neither agree nor disagree
  - Agree
- Shortage in supply of tests prevents me from offering testing to patients
  - Strongly disagree
  - Disagree
  - Neither agree nor disagree
  - Agree
- I know who I should test for **HBV** and **HCV** according to the latest decree issued by the MoH (Decree number 191)
  - Strongly disagree
  - Disagree
  - Neither agree nor disagree
  - Agree
  - Strongly agree
- I know who I should test for **HIV** according to the latest decree issued by the MoH (Decree number 191)
  - Strongly disagree
  - Disagree
  - Neither agree nor disagree
  - Agree
  - Strongly agree
- I need more training to be able to offer **HBV** (HDV) and **HCV** testing
  - Strongly disagree
  - Disagree
  - Neither agree nor disagree
  - Agree
  - Strongly agree
- I need more training to be able to offer **HIV** testing
  - Strongly disagree
  - Disagree
  - Neither agree nor disagree
  - Agree
  - Strongly agree
  - I think it is easier to offer **HBV** testing (compared to HCV and HIV) because of the opportunity to vaccinate for HBV if the test is negative
- Strongly disagree
- Disagree
- Neither agree nor disagree
- Agree
- Strongly agree
- Access to free testing and treatment makes it easier to offer **HBV and HCV** testing
- Strongly disagree
- Disagree
- Neither agree nor disagree
- Agree

Strongly agree

- I think that a lack of free testing and treatment of HDV makes it difficult to refer HBV positive patients to HDV testing
- Strongly disagree
- Disagree
- Neither agree nor disagree
- Agree
- Strongly agree
- Access to free testing and treatment makes it easier to offer **HIV** testing
- Strongly disagree
- Disagree
- Neither agree nor disagree
- Agree
- Strongly agree
- Patients decline to get tested for **HBV and/or HCV** when offered testing in our healthcare facility
- Strongly disagree
- Disagree
- Neither agree nor disagree
- Agree
- Strongly agree
- Patients decline to get tested for **HIV** when offered testing in our healthcare facility
- Strongly disagree
- Disagree
- Neither agree nor disagree
- Agree
- Strongly agree

Key populations

*While strategies for HIV, viral hepatitis and STIs include additional priority populations (such as healthcare workers and pregnant women for HBV), and other populations vulnerable to HIV, five groups, originally defined as “key” in the HIV response have been defined by World Health Organization. These groups are particularly vulnerable to HIV, viral hepatitis and sexually transmitted diseases, and often face different obstacles in reaching healthcare due to social, legal, structural and other contextual factors. Below we would like to ask a few questions about key populations in your healthcare facility.*

- Which of the following key populations are currently patients in your facility?
  - - Gay men and other men who have sex with men
    - People who inject drugs
    - People in prisons and other closed settings
    - Sex workers
    - Trans and gender diverse people
    - None of these groups

|  | If I had a choice, I would prefer not to provide care or services to… | | | | |
| --- | --- | --- | --- | --- | --- |
| People who inject prohibited drugs | Strongly Agree | Agree | Neither agree nor disagree | Disagree | Strongly Disagree |
| Men who have sex with men | Strongly Agree | Agree | Neither agree nor disagree | Disagree | Strongly Disagree |
| Sex workers | Strongly Agree | Agree | Neither agree nor disagree | Disagree | Strongly Disagree |
| Trans and gender diverse people | Strongly Agree | Agree | Neither agree nor disagree | Disagree | Strongly Disagree |

- I prefer not to provide care or services to people who inject prohibited drugs because… (Please check all reasons that apply).
  - - They put me at higher risk for disease
    - This group engages in immoral behaviour
    - I have not received training to work with this group
- I prefer not to provide care or services to men who have sex with men because…

(Please check all reasons that apply).

- - - They put me at higher risk for disease
    - This group engages in immoral behaviour
    - I have not received training to work with this group

- I prefer not to provide care or services to sex workers because…

(Please check all reasons that apply).

- - - They put me at higher risk for disease
    - This group engages in immoral behaviour
    - I have not received training to work with this group
- I prefer not to provide care or services to trans and gender diverse people

(Please check all reasons that apply).

- - - They put me at higher risk for disease
    - This group engages in immoral behaviour
    - I have not received training to work with this group

Hepatitis B Vaccination

*Finally, we would like to ask if you have been vaccinated for hepatitis B. Again, your responses to this question are anonymous and will have no consequences.*

- Have you been vaccinated against hepatitis B?
- Yes
- No
- Do not know
- If yes, when were you vaccinated against hepatitis B?
- More than 10 years ago
- Two to 10 years ago
- Less than two years ago
- If yes, are you fully vaccinated?
- Yes, I received at least three doses
- No, I received only 1 or 2 doses
- I do not remember

**The survey is complete – many thanks for your time and participation!**
