# Supplementary figures and images for: Barriers and facilitators to HIV and viral hepatitis testing in primary healthcare settings in the Kyrgyz Republic: A mixed-methods study using the COM-B Framework
Source: PLoS One. 2025 Nov 17;20(11):e0336257. doi: 10.1371/journal.pone.0336257 (PMC12622783; doi:10.1371/journal.pone.0336257)

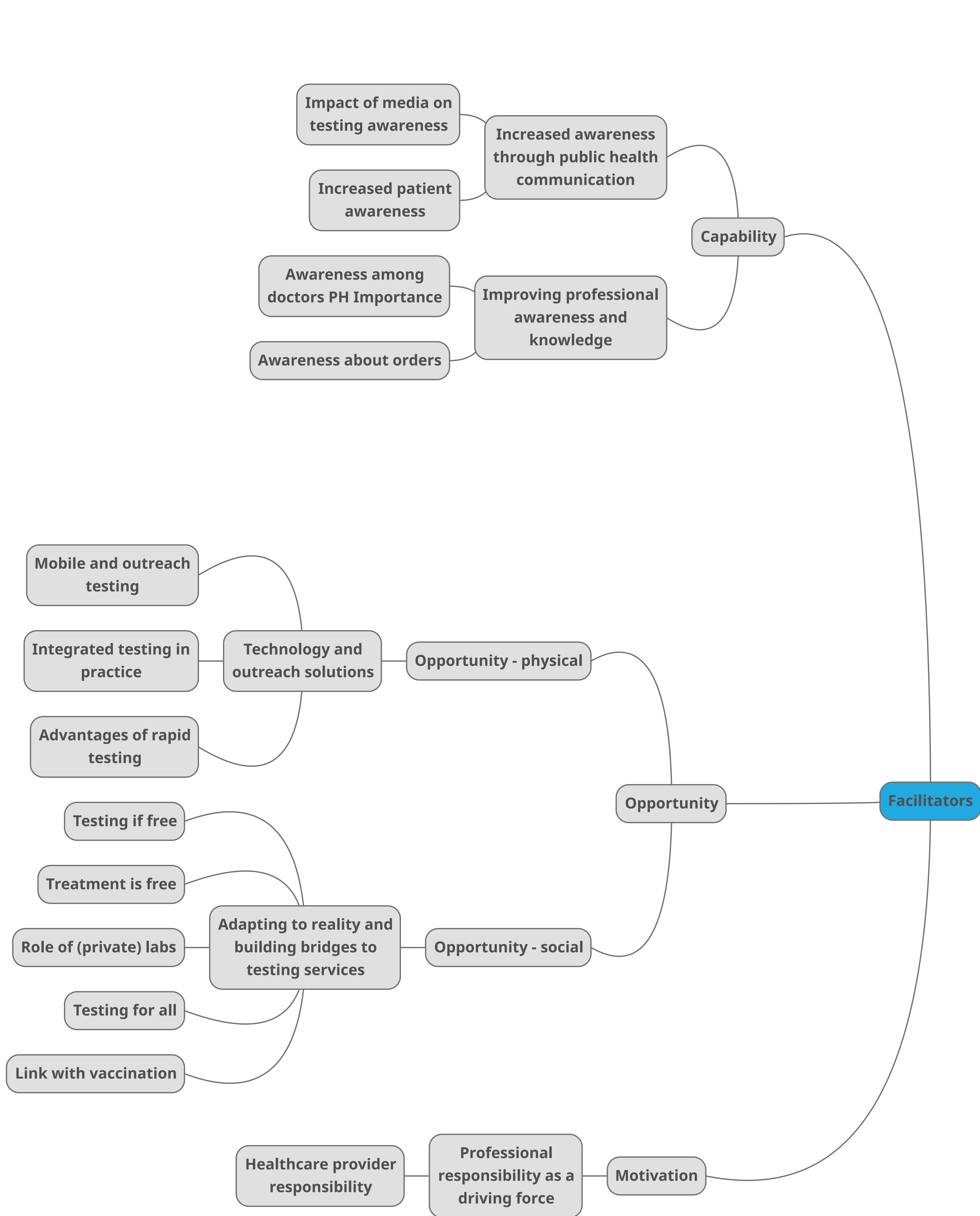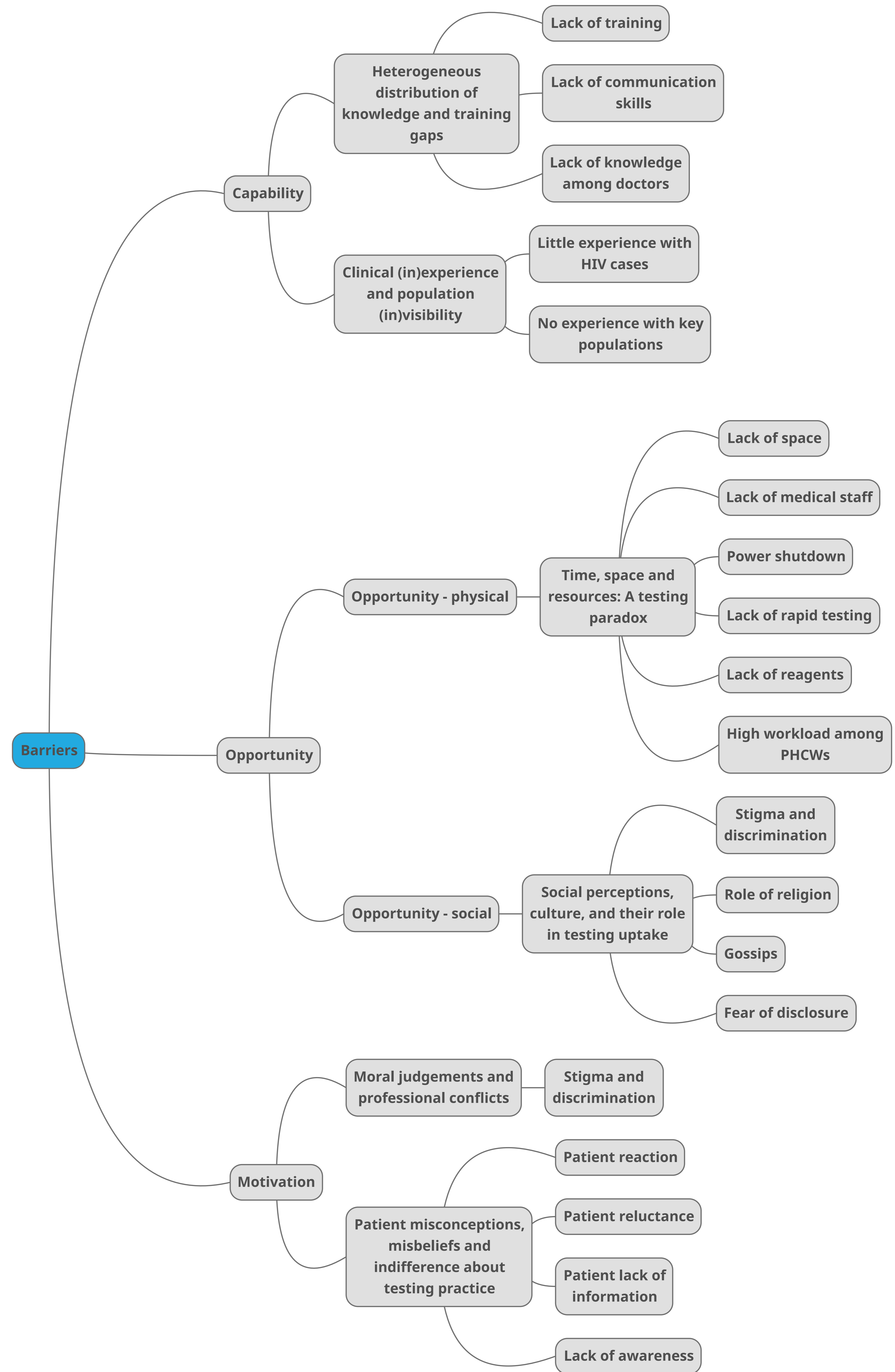

Supplement: S3 Appendix — (PDF) [file pone.0336257.s003.pdf]
