# Supplementary material for: Barriers and facilitators to HIV and viral hepatitis testing in primary healthcare settings in the Kyrgyz Republic: A mixed-methods study using the COM-B Framework
Source: PLoS One. 2025 Nov 17;20(11):e0336257. doi: 10.1371/journal.pone.0336257 (PMC12622783; doi:10.1371/journal.pone.0336257)
Supplement: S4 Appendix — (DOCX) [file pone.0336257.s004.docx]

Appendix 3: Stratified analyses, supplementary tables

**Table 1: Need of more training to offer HBV and HCV testing by age, urban/rural and north/south location of PHC facility**

|  |  | **Strongly disagree n (%)** | **Disagree n (%)** | **Neither agree nor disagree**  **n (%)** | **Agree**  **n (%)** | **Strongly Agree**  **n (%)** | **No reply (missing)**  **n (%)** | **p-value*** |
| --- | --- | --- | --- | --- | --- | --- | --- | --- |
| **Age** | 18-50 years | 16 (2.9) | 108 (20) | 56 (10) | 281 (51) | 67 (12) | 19 (3.5) | 0.4 |
|  | 51-61+ years | 16 (3.2) | 114 (23) | 37 (7.3) | 263 (52) | 58 (12) | 18 (3.6) |  |
| **Urban/rural** | Primary healthcare facility located in the city (urban) | 17 (3.0) | 110 (19) | 49 (8.6) | 304 (53) | 73 (13) | 18 (3.2) | 0.5 |
|  | Primary healthcare facility located in the village (rural) | 17 (3.5) | 114 (23) | 43 (8.8) | 247 (50) | 53 (19) | 17 (3.5) |  |
| **North/South** | Primary healthcare facility located in the South | 10 (2.9) | 69 (20) | 25 (7.2) | 184 (53) | 45 (13) | 12 (3.5) | 0.6 |
|  | Primary healthcare facility located in the North | 24 (3.3) | 156 (22) | 68 (9.4) | 368 (51) | 81 (11) | 23 (3.2) |  |

* Pearson’s Chi-squared test

**Table 2: Lack of free Need of free testing and treatment of HDV makes it difficult to refer HBV positive patients to HDV testing by age, urban/rural and north/south location of PHC facility**

|  |  | **Strongly disagree n (%)** | **Disagree n (%)** | **Neither agree nor disagree**  **n (%)** | **Agree**  **n (%)** | **Strongly Agree**  **n (%)** | **No reply (missing)**  **n (%)** | **p-value*** |
| --- | --- | --- | --- | --- | --- | --- | --- | --- |
| **Age** | 18-50 years | 13 (2.4) | 122 (22) | 60 (11) | 276 (51) | 58 (11) | 18 (3.3) | 0.4 |
|  | 51-61+ years | 20 (4.0) | 131 (26) | 38 (7.5) | 244 (48) | 54 (11) | 19 (3.8) |  |
| **Urban/rural** | Primary healthcare facility located in the city (urban) | 23 (4.0) | 140 (25) | 59 (10) | 270 (47) | 56 (9.8) | 23 (4.0) | 0.5 |
|  | Primary healthcare facility located in the village (rural) | 23 (4.3) | 108 (22) | 60 (12) | 233 (48) | 49 (10) | 20 (4.1) |  |
| **North/South** | Primary healthcare facility located in the South | 16 (4.6) | 79 (23) | 23 (6.7) | 174 (50) | 37 (11) | 16 (4.6) | 0.2 |
|  | Primary healthcare facility located in the North | 28 (3.9) | 169 (24) | 97 (14) | 331 (46) | 68 (9.4) | 27 (3.8) |  |

* Pearson’s Chi-squared test

**Table 3: Need of more training to offer HIV testing by age, urban/rural and north/south location of PHC facility**

|  |  | **Strongly disagree n (%)** | **Disagree n (%)** | **Neither agree nor disagree**  **n (%)** | **Agree**  **n (%)** | **Strongly Agree**  **n (%)** | **No reply (missing)**  **n (%)** | **p-value*** |
| --- | --- | --- | --- | --- | --- | --- | --- | --- |
| **Age** | 18-50 years | 13 (2.4) | 122 (22) | 60 (11) | 276 (51) | 58 (11) | 18 (3.3) | 0.1 |
|  | 51-61+ years | 20 (4.0) | 131 (26) | 38 (7.5) | 244 (48) | 54 (11) | 19 (3.8) |  |
| **Urban/rural** | Primary healthcare facility located in the city (urban) | 17 (3.0) | 127 (22) | 48 (8.4) | 293 (51) | 67 (12) | 19 (3.3) | 0.3 |
|  | Primary healthcare facility located in the village (rural) | 17 (3.5) | 127 (26) | 49 (10) | 235 (48) | 47 (9.6) | 16 (3.3) |  |
| **North/South** | Primary healthcare facility located in the South | 9 (2.6) | 75 (22) | 31 (9.0) | 175 (51) | 43 (13) | 12 (3.5) | 0.4 |
|  | Primary healthcare facility located in the North | 25 (3.5) | 180 (25) | 67 (9.3) | 355 (49) | 70 (9.7) | 23 (3.2) |  |

* Pearson’s Chi-squared test
